# Surgical Techniques at Cesarean Delivery: A U.S. Survey

**DOI:** 10.1055/s-0036-1594247

**Published:** 2016-11-14

**Authors:** Deirdre J. Lyell, Michael Power, Katie Murtough, Amen Ness, Britta Anderson, Kristine Erickson, Jay Schulkin

**Affiliations:** 1 Department of Obstetrics and Gynecology, Stanford University, Palo Alto, California; 2 Research Department, American College of Obstetricians & Gynecologists, Washington, District of Columbia

**Keywords:** cesarean, survey, surgical technique, surgical closure

## Abstract

**Objective** To assess the frequency of surgical techniques at cesarean delivery (CD) among U.S. obstetricians.

**Methods** Members of the American College of Obstetrician Gynecologists were randomly selected and e-mailed an online survey that assessed surgical closure techniques, demographics, and reasons. Data were analyzed using SPSS (IBM Corp., Armonk, New York, United States), descriptive statistics, and analysis of variance.

**Results** Our response rate was 53%, and 247 surveys were analyzed. A similar number of respondents either “always or usually” versus “rarely or never” reapproximate the rectus muscles (38.4% versus 43.3%, *p* = 0.39), and close parietal peritoneum (42.5% versus 46.9%, *p* = 0.46). The most frequently used techniques were double-layer hysterotomy closure among women planning future children (73.3%) and suturing versus stapling skin (67.6%); the least frequent technique was closure of visceral peritoneum (12.2%). Surgeons who perform double-layer hysterotomy closure had fewer years in practice (15.0 versus 18.7 years, *p* = 0.021); surgeons who close visceral peritoneum were older (55.5 versus 46.4 years old, *p* < 0.001) and had more years in practice (23.8 versus 13.8 years practice; *p* < 0.001).

**Conclusion** Similar numbers of obstetricians either reapproximate or leave open the rectus muscles and parietal peritoneum at CD, suggesting that wide variation in practice exists. Surgeon demographics and safety concerns play a role in some techniques.

Following a nearly continuous rise since 1996, cesarean delivery (CD) remains the most common major surgery performed among women in the United States, at a rate of 32.8% in 2012. 1 Despite the high frequency of CD, few high-quality data exist regarding the effects of different surgical techniques on maternal or neonatal morbidity, or the frequency of their use. Some surgical closure techniques at CD, such as closure of the parietal and visceral peritoneum, have been shown to influence short-term outcomes such as increased operative time, 2 increased infectious morbidity with closure of the visceral peritoneum, 3 and long-term outcomes such as adhesions and their consequences. 4 5 6 7 8 Techniques such as rectus muscle reapproximation are not typically mentioned in studies, 9 10 11 and it is unknown how frequently they are used at CD.

Anecdotal evidence suggests that a large variation in surgical closure techniques exists among obstetricians, particularly for techniques such as rectus muscle reapproximation. A European survey identified that a large practice variation exists for most surgical techniques used at CD, with the exception of double-layer hysterotomy closure, a technique that was practiced by most. 12 A recently published Canadian survey of hysterotomy closure techniques at CD also revealed a high frequency of double-layer hysterotomy closure. 13 Although a growing number of randomized controlled trials have been published regarding surgical techniques at CD and short-term outcomes, 9 11 little is known about what obstetricians actually do in practice in the United States. It is important to identify which surgical techniques are used at CD in current practice, in concert with studies that identify which techniques should be used in best practice.

We surveyed U.S. obstetricians to assess the frequency of surgical closure techniques used at CD, and identified the variation in practice and the reasons for choosing each technique.

## Methods

One thousand members of American College of Obstetrician Gynecologists (ACOG) were randomly selected and invited to participate in this study between April and June of 2014. Two hundred of the participants were members of the Collaborative Ambulatory Research Network (CARN), a network of ACOG members who have agreed to participate in research conducted by ACOG. Participants were contacted by e-mail with basic information about the study and an electronic link to access the survey. E-mail recipients who do not practice obstetrics were given a link to opt out. Obstetricians who perform CD and completed at least 60% of the survey were included. A total of six reminder e-mails were sent to those who had not opened or responded to the survey, each approximately within 1 week of each other. A follow-up letter was sent via mail to those who had not opened any of the e-mails and to those who had opened an e-mail but had not yet responded ( *n* = 699) at the time of the fifth e-mail reminder.

**Fig. 1:**
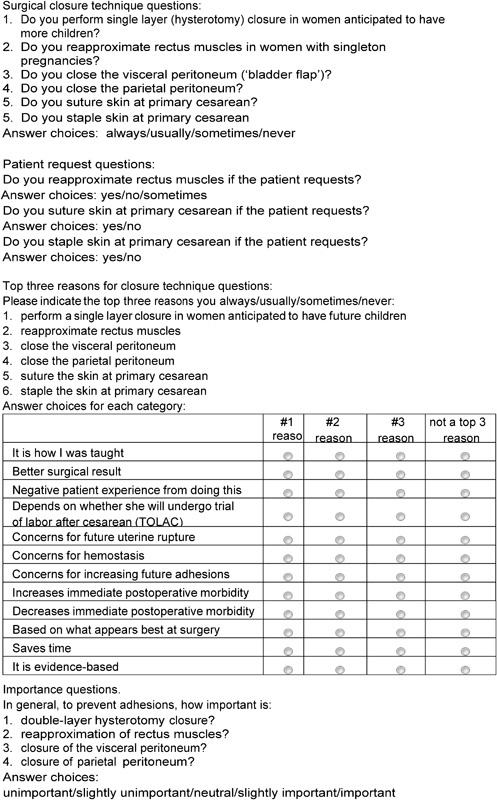
Summary of survey questions.

The survey consisted of 19 routine questions about respondent demographics and 25 questions about surgical techniques. The surgical techniques questions are summarized in Fig. 1 .

CARN Fellows (1,248 members as of February 2015) are a group of ACOG Fellows and Junior Fellows in Practice (JFPs) who volunteer to participate in survey studies on an ongoing basis. CARN Fellows are a nationally representative sample of the age, sex, and geographic distribution of ACOG Fellows and JFPs, with proportionate representation from all 11 ACOG districts. Assessments of knowledge and practice between CARN Fellows and ACOG Fellows not in the CARN have shown they do not differ. An examination of 17 randomly selected studies showed few differences in responses between CARN and non-CARN ACOG study participants, with a mean correlation of *r* = 0.99 ( *p* < 0.001) for the responses to ordinal questions (e.g., 1- to 5-point scales). Although ACOG cannot exclude the possibility that CARN Fellows differ in aspects of knowledge, training, or practice in some areas of clinical medicine, to date that has not been shown to be true. ACOG continues to test for differences every year.

Data were analyzed using IBM SPSS Statistics 20.0 (IBM Corp., Armonk, New York, United States). For the purposes of data analysis, the responses “always” and “usually” were combined and considered to be commonly performed surgical techniques, and “rarely” and “never” were combined and considered to be techniques not commonly performed. Chi-squared and Fisher exact tests were used where relevant, and analysis of variance analyses were used to measure group differences; *p* < 0.05 was considered statistically significant.

This study was reviewed by the Institutional Review Board and Stanford University and was determined to be exempt.

## Results

Of the 1,000 obstetrician-gynecologists invited to participate, 48 did not have valid e-mail addresses. Of the remaining 952, 706 opened at least one e-mail and/or confirmed receipt of the follow-up letter; 146 responded that they do not perform CDs or had retired from practice and were considered ineligible to participate. The final sample included 560 potentially eligible responders, of whom 295 participated in the online survey for a 53% total response rate (70% CARN, 48% non-CARN). Of the 295 responses, 48 were incomplete, defined by less than 60% of the survey completed. Data analyses reflect the responses of 247 participants (44% complete response rate).

Demographics for the survey respondents are shown in Table 1 . The cohort was 63.6% women, 86.6% generalist obstetrician-gynecologists, with an average age of 48.4 (standard error of the mean, ± 0.7), and average years in practice of 16.1 (standard error of the mean, ± 0.7). The largest group (37%) were from large (≥5 surgeons) practices, 18% were from university or teaching practices, and 12% were in solo practice.

**Table 1:** Demographics of respondents a

		*n*
Male (%)	36.4	90
Female (%)	63.6	157
Average age (y; mean ± SEM)	48.4 ± 0.7	246
Average years in practice (mean ± SEM)	16.1 ± 0.7	246
Generalist (%)	86.6	214
Maternal–fetal medicine (%)	6.9	17
Small (2–4) private partnership (%)	27.5	68
Large (≥5) private partnership (%)	37.2	92
Solo practice (%)	11.7	29
Laborist/hospitalist (%)	3.6	9
University/teaching institution (%)	18.2	45
More than 50% private insurance in practice (%)	58.3 ± 1.9	239
More than 50% public insurance in practice (%)	32.7 ± 1.6	239
More than 50% uninsured in practice (%)	5.2 ± .5	239

a Percent or mean ± SEM where indicated. *n* refers to total respondents in each group, from a total of 247.

SEM, standard error of the mean.

The frequencies of surgical techniques used are shown in Table 2 . The most frequently used techniques were double-layer hysterotomy closure among women planning future children (73.3%), and suturing of the skin (67.6%); the least frequently used technique was closure of the visceral peritoneum (12.2%). Wide variation was seen in the techniques of rectus muscle reapproximation and closure of the parietal peritoneum, with similar numbers reporting that they “always or usually” versus “rarely or never” reapproximate the rectus muscles (38.4% versus 43.3%, *p* = 0.39), and “always or usually” versus “rarely or never” close the parietal peritoneum (42.5% versus 46.9%, *p* = 0.46).

**Table 2:** Frequencies of reported surgical techniques, n (%)

	“Always” or “Usually”	“Sometimes”	“Rarely” or “Never”	No answer
Single-layer hysterotomy closure if more children planned	39 (15.8)	22 (8.9)	181 (73.3)	2 (2.0)
Close the parietal peritoneum	105 (42.5)	23 (9.3)	116 (46.9)	3 (1.2)
Close the visceral peritoneum	30 (12.2)	14 (5.7)	194 (78.5)	9 (3.6)
Reapproximate the rectus muscles (singletons)	95 (38.4)	41 (16.6)	107 (43.3)	4 (1.6)
Suture the skin	167 (67.6)	31 (12.6)	43 (17.4)	6 (2.4)
Staple the skin	56 (22.7)	39 (15.8)	145 (58.7)	7 (2.8)

Demographic differences were identified based on practice choices ( Table 3 ). Surgeons who were more likely (“always” or “usually”) to perform a single-layer hysterotomy closure for women planning future children had more years in practice compared with surgeons who were less likely (“rarely” or “never”) to do so (18.7 versus 15.0 years in practice, *p* = 0.02). Surgeons who were more likely to close the parietal peritoneum were older, with more years in practice, compared with surgeons who were less likely to do so (50.0 versus 46.5 years old, *p* = 0.009;17.6 versus 14.1 years in practice, *p* = 0.012), and similar patterns were seen among those who close the visceral peritoneum (55.5 versus 46.4 years old, *p* < 0.001; 23.8 versus 13.8 years in practice, *p* < 0.001) and among those who reapproximate the rectus muscles (49.4 versus 46.8 years old, *p* = 0.052; 17.2 versus 14.5 years in practice, *p* = 0.055). Surgeons who were more likely to suture the skin had fewer years in practice (15.1 versus 19.2 years, *p* = 0.022), and no differences were found among those who were more likely to staple the skin.

**Table 3:** Surgical techniques by average respondent age and years in practice a

	“Always” or “Usually”	“Sometimes”	“Rarely” or “Never”	*p* Value b
Perform a single-layer hysterotomy				
Age	49.7 ± 1.7 (38)	50.6 ± 3.1 (22)	47.7 ± 0.7 (181)	0.328
Years in practice	18.5 ± 1.7 (39)	19.0 ± 3.1 (22)	15.0 ± 0.8 (180)	0.069
Close the parietal peritoneum				
Age	50.3 ± 1.0 (105)	48.7 ± 2.0 (23)	46.5 ± 1.0 (115)	0.028
Years in practice	18.1 ± 1.1 (105)	15.5 ± 2.2 (22)	14.1 ± 1.0 (116)	0.025
Close the visceral peritoneum				
Age	55.7 ± 1.4 (30)	55.0 ± 2.9 (14)	46.4 ± 0.7 (193)	<0.001
Years in practice	24.3 ± 1.4 (30)	22.9 ± 3.1 (14)	13.8 ± 0.7 (193)	<0.001
Reapproximate the rectus muscles				
Age	50.7 ± 1.0 (95)	46.6 ± 1.5 (41)	46.8 ± 1.1 (106)	0.016
Years in practice	18.3 ± 1.1 (95)	14.4 ± 1.6 (40)	14.5 ± 1.1 (107)	0.023
Suture the skin				
Age	47.7 ± 0.8 (167)	47.7 ± 2.3 (31)	51.0 ± 1.4 (42)	0.174
Years in practice	15.1 ± 0.8 (166)	15.3 ± 2.4 (31)	19.3 ± 1.5 (43)	0.072
Staple the skin				
Age	50.9 ± 1.4 (55)	47.3 ± 1.9 (39)	47.7 ± 0.8 (145)	0.121
Years in practice	18.8 ± 1.5 (56)	14.4 ± 1.8 (39)	15.2 ± 0.9 (144)	0.066

a Years, mean ± standard error of the mean, and ( *n* ) where indicated.

b *p* Values were calculated using analysis of variance.

We asked surgeons to list their top three reasons for choosing each technique. “Evidence-based” was cited in the top three for a surgeon's choice regarding single-layer hysterotomy closure, visceral peritoneum closure, and suturing of the skin ( Table 4 ). Surgeons who chose “evidence-based” as a top three reason for their practice were less likely to close the parietal peritoneum (75.9%) or visceral peritoneum (98.3%). “It was how I was taught” was frequently a top three reason, cited for a surgeon's decision regarding his or her choice for single-layer hysterotomy closure, and decisions to close the parietal peritoneum, visceral peritoneum, and rectus muscles, without any single technique being performed more frequently. Surgeons who chose “better surgical result” as a top three reason were more likely to close the parietal peritoneum (71.6% versus 17.1% who did not give this response as a reason, *p* < 0.001) and reapproximate rectus muscles (61.8% versus 6.1% who did not give this response as a reason ( *p* < 0.001)). What “Appears best at time of surgery” was frequently cited for rectus muscle reapproximation and skin closure techniques. “Concerns for increasing future adhesions” was the top choice among those who close the parietal peritoneum, the only situation in which this response appears as a top three choice.

**Table 4:** Reasons for using specific surgical techniques, *n* (%)

	Most frequent response	Second most frequent	Third most frequent	Least frequent
Double-layer hysterotomy closure if future children planned	“Concern for future uterine rupture,” 174 (70.4)	“Evidence-based,” 119 (48.2)	“It was how I was taught,” 104 (42.1)	“Increases immediate postoperative morbidity,” 4 (1.6)
Close parietal peritoneum	“Concerns for increasing future adhesions,” 134 (54.3)	“It was how I was taught,” 129 (52.2)	“Better surgical result,” 117 (47.4)	“Increases immediate postoperative morbidity,” 11 (4.5)
Close visceral peritoneum	“It was how I was taught,” 134 (54.3)	“Saves time,” 131 (53.0)	“Evidence-based,” 117 (47.4)	“Decreases immediate postoperative morbidity,” 17 (6.9)
Reapproximate rectus muscles (singletons)	“Appears best at time of surgery,” 146 (59.1)	“Better surgical result,” 145 (58.7)	“It was how I was taught,” 118 (47.8)	“Decreases immediate postoperative morbidity,” 16 (6.5)
Suture skin	Tie: “Better cosmetic result” and “Patient preference,” 159 (64.4)	Tie: “Better cosmetic result” and “Patient preference,” 159 (64.4)	“Evidence-based,” 81 (32.8)	“Increases immediate postoperative morbidity,” 3 (1.2)
Staple skin	“Saves time,” 134 (54.3)	“Appears best at time of surgery,” 113 (45.7)	“Better cosmetic result,” 83 (33.6)	“Increases immediate postoperative morbidity,” 21 (8.5)

The most frequently chosen response about a specific surgical technique overall was not to perform single-layer hysterotomy closure among women planning future children, something that 73.3% reported they were less likely to perform ( Table 2 ); 70.4% of surgeons reported choosing their answer based on concern for future uterine rupture, with “Evidence-based” as the second most frequent response for this technique ( Table 4 ). Suturing of the skin due to both better cosmetic result and patient preference were the next most frequently chosen reasons (159 responses; 64.4% each).

When asked to choose from a given list, the most important techniques to prevent adhesions, surgeons reported the following in order of frequency: parietal peritoneum closure (42.2%), double-layer hysterotomy closure (29.6%), reapproximation of the rectus muscles (19.9%), and visceral peritoneum closure (13.4%).

## Discussion

Our study demonstrates a lack of consistency in the practices of rectus muscle reapproximation and parietal peritoneum closure at CD among survey respondents in the United States, with similar numbers of obstetricians practicing each technique (38% reapproximate rectus muscles, 43% do not; 43% close the parietal peritoneum, 47% do not). In contrast, we found that double-layer hysterotomy closure and suturing of the skin are commonly practiced, and closure of the visceral peritoneum is uncommon. Significant differences were seen in practice styles based on age and years in practice. Older doctors with more years in practice were more likely to close the parietal peritoneum and the visceral peritoneum and to reapproximate the rectus muscles; doctors with fewer years in practice were more likely to close the hysterotomy in two layers and to suture the skin. Most surgeons report that their practice style reflects primarily how they were trained.

Evidence-based guidelines exist for some, but not all, of the surgical closure techniques we queried. Dahlke et al updated a systematic review from Berghella et al, 9 11 including randomized controlled trials, meta-analyses or systematic reviews, and Cochrane reviews, most of which focus on short-term outcomes, and made evidence-based recommendations using the U.S. Preventative Services Task Force definitions. No recommendation could be made regarding rectus muscle reapproximation due to insufficient evidence. Indeed, no randomized controlled trials of rectus reapproximation have been published. When the search terms “rectus,” “muscle,” and “cesarean” or “rectus,” “cesarean,” and “closure” were entered into PubMed, utilizing the English language literature from 1950 to the present, only one result about rectus muscle reapproximation was returned, 6 a prospective cohort study that suggested a decrease in adhesions with reapproximation. Given the limited data about rectus muscle reapproximation, the lack of consistency in practice styles is likely to continue.

Data are limited regarding optimal surgical techniques at CD to reduce postsurgical adhesions, 6 given the difficulty of conducting a long-term CD follow-up study. With regard to peritoneal closure, several studies compared a combined closure of the parietal and visceral peritoneum to nonclosure, 14 15 16 17 so may not answer the question of peritoneum closure and adhesions, as some studies suggest that closure of the parietal peritoneum may decrease adhesions while closure of the visceral peritoneum may increase them. 4 6 18 Dahlke et al concluded that based on limited data, parietal peritoneal closure may decrease the risk of future adhesions. 9

Few (16%) of respondents reported routinely closing the hysterotomy in one-layer among women planning future children, and 73% reported rarely taking this approach. In their systematic review, Dahlke et al acknowledge that data on uterine rupture are derived from cohort and case control studies, not randomized controlled trials. 9 Two randomized controlled trials examining this question, the CORONIS and CAESAR collaboratives, are currently ongoing. 14 15 Without a randomized controlled trial, the majority of U.S. obstetricians have chosen a more conservative approach and practice double-layer hysterotomy closure. Of note, we did not query respondents about their hysterotomy closure technique for women not planning future children as the question of uterine rupture was the factor underlying our question.

The majority of respondents (68%) typically suture the skin, style unspecified, compared with 23% who typically use staples. According to the systematic review by Dahlke et al, 9 staple closure was associated with a twofold greater risk of wound infection or separation compared with subcuticular closure, though a Cochrane review concluded that wound complications were similar with each technique. 19 Based on this difference, Dahlke et al concluded that a definitive recommendation is difficult due to uncertainty. 9

Two similar surveys of surgical techniques at CD conducted by Demers et al and Tully et al also demonstrated significant variation in closure techniques. 12 13 In a survey of 176 obstetrician-gynecologists conducted in Quebec, Canada, respondents similarly reported a lack of consistency in the practice closure of the parietal peritoneum (49% close, 50% do not close), reported that most do not close the visceral peritoneum (17% close, 81% do not close) and that most close the hysterotomy in two layers (89% versus 10% single layer). 13 In contrast to our results, fewer respondents reapproximate the rectus muscles (27% close, 72% do not close), and more close the skin with staples (86% versus 14% use suture). In a 1999 survey of 2,374 members of the Royal College of Obstetricians and Gynaecologists, Tully et al found that, compared with our results, fewer surgeons closed the parietal peritoneum (34% versus 66% did not close), more closed the visceral peritoneum (28% versus 72% did not close), and nearly all closed the hysterotomy in two layers (94% versus 3% single layer); rectus muscle reapproximation was not mentioned. 13

Our study has several strengths. Our survey was sent to a large number of ACOG members, included practitioners from all practice environments, and should be reflective of contemporary practice. In contrast to published systematic reviews on evidence-based surgical techniques at CD, our study addresses a unique question that is otherwise to date unaddressed in the United States: what surgical practices do obstetricians actually employ at CD? Our study is limited by potentially inherent and difficult to measure biases reflected in any survey, such as whether there may be practice differences among those who chose to respond, and differences among those who use e-mail, as those not using e-mail were not included. We sent the survey to many who no longer practice obstetrics, as there was no way to identify in those who do not practice.

CD is the most common surgical procedure performed in women in the United States, yet similar to surveys conducted in Canada and Britain, our survey demonstrated significant variation in surgical closure techniques. Age, years in practice, and how one was trained are some of the reasons for such variation. The lack of a strong evidence base for techniques to minimize long-term, and in some cases short-term, morbidity likely also plays a role in the observed practice variation. The prospective randomized trials currently being conducted through the CORONIS and CAESAR collaboratives may answer some questions about optimal surgical techniques, particularly with regard to hysterotomy closure. 14 15

Given the limited level 1evidence for many closure techniques at CD, it is likely current practices will remain unchanged until a stronger evidence base exists.

## References

[1] Martin J A Hamilton B E Osterman M JK Births: final data for 2012. National Vital Statistics Reports. Vol. 62. No. 9 Hyattsville, MD National Center for Health Statistics 2013 25671704

[2] Irion O Luzuy F Béguín F Nonclosure of the visceral and parietal peritoneum at caesarean section: a randomised controlled trial Br J Obstet Gynaecol 1996 103 07 690 694 8688397 10.1111/j.1471-0528.1996.tb09839.x

[3] Nagele F Karas H Spitzer D Closure or nonclosure of the visceral peritoneum at cesarean delivery Am J Obstet Gynecol 1996 174 04 1366 1370 8623871 10.1016/s0002-9378(96)70686-5

[4] Lyell D J Caughey A B Hu E Daniels K Peritoneal closure at primary cesarean delivery and adhesions Obstet Gynecol 2005 106 02 275 280 16055575 10.1097/01.AOG.0000171120.81732.4c

[5] Lyell D J Adhesions and perioperative complications of repeat cesarean delivery Am J Obstet Gynecol 2011 205(6, Suppl):S11 S18 22114993 10.1016/j.ajog.2011.09.029

[6] Lyell D J Caughey A B Hu E Blumenfeld Y El-Sayed Y Y Daniels K Rectus muscle and visceral peritoneum closure at cesarean delivery and intraabdominal adhesions Am J Obstet Gynecol 2012 206 06 5150 5.15E7 10.1016/j.ajog.2012.02.033 22463952

[7] Blumenfeld Y J Caughey A B El-Sayed Y Y Daniels K Lyell D J Single- versus double-layer hysterotomy closure at primary caesarean delivery and bladder adhesions BJOG 2010 117 06 690 694 20236104 10.1111/j.1471-0528.2010.02529.x

[8] Greenberg M B Daniels K Blumenfeld Y J Caughey A B Lyell D J Do adhesions at repeat cesarean delay delivery of the newborn? Am J Obstet Gynecol 2011 205 04 3800 3.8E7 10.1016/j.ajog.2011.06.088 21864825

[9] Dahlke J D Mendez-Figueroa H Rouse D J Berghella V Baxter J K Chauhan S P Evidence-based surgery for cesarean delivery: an updated systematic review Am J Obstet Gynecol 2013 209 04 294 306 23467047 10.1016/j.ajog.2013.02.043

[10] Hofmeyr J G Novikova N Mathai M Shah A Techniques for cesarean section Am J Obstet Gynecol 2009 201 05 431 444 19879392 10.1016/j.ajog.2009.03.018

[11] Berghella V Baxter J K Chauhan S P Evidence-based surgery for cesarean delivery Am J Obstet Gynecol 2005 193 05 1607 1617 16260200 10.1016/j.ajog.2005.03.063

[12] Tully L Gates S Brocklehurst P McKenzie-McHarg K Ayers S Surgical techniques used during caesarean section operations: results of a national survey of practice in the UK Eur J Obstet Gynecol Reprod Biol 2002 102 02 120 126 11950477 10.1016/s0301-2115(01)00589-9

[13] Demers S Roberge S Afiuni Y A Chaillet N Girard I Bujold E Survey on uterine closure and other techniques for Caesarean section among Quebec's obstetrician-gynaecologists J Obstet Gynaecol Can 2013 35 04 329 333 23660040 10.1016/S1701-2163(15)30960-9

[14] The CORONIS Collaborative Group.CORONIS–International study of caesarean section surgical techniques: the follow-up study BMC Pregnancy and Childbirth 2013 13 215 24261693 10.1186/1471-2393-13-215 PMC4222281

[15] CAESAR study collaborative group.Caesarean section surgical techniques: a randomised factorial trial (CAESAR) BJOG 2010 117 11 1366 1376 20840692 10.1111/j.1471-0528.2010.02686.x

[16] Roset E Boulvain M Irion O Nonclosure of the peritoneum during caesarean section: long-term follow-up of a randomised controlled trial Eur J Obstet Gynecol Reprod Biol 2003 108 01 40 44 12694968 10.1016/s0301-2115(02)00366-4

[17] Ghahiry A Rezaei F Karimi Khouzani R Ashrafinia M Comparative analysis of long-term outcomes of Misgav Ladach technique cesarean section and traditional cesarean section J Obstet Gynaecol Res 2012 38 10 1235 1239 22845718 10.1111/j.1447-0756.2011.01777.x

[18] Woytoń J Florjański J Zimmer M [Nonclosure of the visceral peritoneum during Cesarean sections] Ginekol Pol 2000 71 10 1250 1254Polish. 11143933

[19] Mackeen A D Berghella V Larsen M L Techniques and materials for skin closure in caesarean section Cochrane Database Syst Rev 2012 11 CD003577 22972064 10.1002/14651858.CD003577.pub2

